# Exclusive enteral nutrition with concomitant early thiopurine use was effective in maintaining steroid-free remission in a Southeast Asian cohort of children with Crohn’s disease

**DOI:** 10.1186/s12876-018-0907-7

**Published:** 2018-12-12

**Authors:** Christina Ong, Poh Ting Lim, Veena Logarajah, Maria Janelle Liwanag, Bi Xia Ang, Yuqin Cher, Fang Kuan Chiou, Ajmal Kader

**Affiliations:** 1 Gastroenterology Service, Department of Paediatric Medicine, KK Women’s and Children’s Hospital, KK Hospital, 100 Bukit Timah Road, Singapore, 229899 Singapore; 2 Yong Loo Lin School of Medicine, National University of Singapore, Singapore, Singapore; 3 Department of Nutrition and Dietetics, KK Women’s and Children’s Hospital, Singapore, Singapore

**Keywords:** Paediatric, Crohn’s disease, Exclusive enteral nutrition, Thiopurines

## Abstract

**Background:**

Exclusive enteral nutrition (EEN) is as effective as corticosteroids in inducing remission in children with Crohn’s disease (CD). However, over 50% of these children relapse by 12 months of diagnosis. Thiopurines are commonly prescribed as maintenance therapy for CD, but evidence for its efficacy is controversial. Data on the effectiveness of EEN in Southeast Asian (SEA) children with CD is scarce. This study aims to evaluate the efficacy of EEN induction therapy in a cohort of SEA children with newly diagnosed CD. The secondary aim was to evaluate concomitant early azathioprine (EAZ) use in determining remission rate at 6 and 12 months.

**Methods:**

Case records of all children with newly diagnosed CD from 2011 to 2014 were reviewed and relevant demographic as well as clinical data were extracted. The primary outcome measure was the number of patients who completed EEN induction therapy and achieved remission (Paediatric Crohn’s Disease Activity Index; PCDAI≤10). Factors influencing duration of remission were evaluated in particular early azathioprine (EAZ) defined as starting azathioprine within one month of diagnosis versus late azathioprine (LAZ) use.

**Results:**

Forty children with newly diagnosed CD were identified. Thirty-three children: 67% boys, median age 13y (range 3–17) completed 8 weeks of EEN induction therapy and 91% achieved remission. Significant improvements were seen in PCDAI scores (32.7 ± 9.2 to 4.2 ± 5.1; *p* < 0.001), mean BMI z-score (− 1.38 ± 1.57 to − 0.82 ± 1.27; *p* = 0.004) and baseline inflammatory markers: Erythrocyte Sedimentation Rate (51.6 ± 30.1 mm/h to 13.3 ± 7.1 mm/h; *p* < 0.0001) C-Reactive Protein (44.6 ± 51.0 mg/L to 5.2 ± 7.6 mg/L; *p* = 0.001), Albumin (30.7 ± 7.5 g/L to 38.7 ± 3.9 g/L; *p* < 0.0001), Platelets (464 ± 161 × 10^9^ to 370 ± 111 × 10^9^; *p* < 0.0001),. Early azathioprine initiation was associated with a remission rate of 80 and 73% at 6 and 12 months respectively. Remission was also maintained for longer duration in EAZ vs LAZ groups (*p* = 0.048).

**Conclusion:**

EEN effectively induces remission in this cohort of SEA children with newly diagnosed CD. Early initiation of thiopurine with EEN induction therapy is effective in maintaining steroid-free remission for at least one year.

## Background

Approximately 25% of Crohn’s disease (CD) cases are diagnosed during childhood and adolescence [1]. The incidence of children with CD is rising and is considered to have a more aggressive phenotype compared to adult-diagnosed CD [1, 2]. Up to 85% of children with CD have weight loss, growth failure, and nutritional impairment at presentation [3, 4]. Growth impairment further leads to pubertal delay and ultimately impacts the acquisition of adult height. Hence, it is vital that nutritional support, as well as growth preservation, remain a central focus in the management of children with CD.

The efficacy of exclusive enteral nutrition (EEN) in achieving remission in active CD is well documented in the western literature and is comparable with corticosteroids [5, 6]. The obvious advantage of EEN is to avoid corticosteroid use since a significant proportion of children with CD already have faltering growth at presentation. In addition, EEN provides nutritional support and gut rehabilitation [7]. Both polymeric and elemental diets have been shown to be effective in inducing remission in CD [8, 9]. Due to its excellent safety profile, the ECCO/ESPGHAN guideline recommends EEN as a therapy of choice for induction of remission in children and adolescents who have not completed their growth as it is preferable over corticosteroids [10].

The mechanisms of EEN therapy are mainly in the modulation of the intestinal microbiome, bowel rest through a reduction in antigenic load, decreased gut metabolic activity and direct anti-inflammatory effects [11, 12]. Despite the evidence, EEN has not been universally accepted in paediatric centres and its efficacy has not been well described in Asian or Southeast Asian (SEA) populations.

Although it is possible to achieve remission with either corticosteroids or EEN, the majority of patients relapse within 12 months. Among patients treated with an elemental diet, over 80% relapse within one year [13, 14]. Similarly, 66% of patients who achieved remission with prednisolone therapy relapse within 18 months after discontinuation of steroid therapy [13]. Sustained remission remains a difficult therapeutic challenge. Studies on the effectiveness of thiopurines as maintenance therapy in Crohn’s disease show conflicting results, with some studies reporting excellent efficacy while others describing its use as no more effective than placebo [15–18].

The aim of this study was to evaluate the efficacy of EEN induction therapy in a cohort of SEA children with newly diagnosed active CD. The secondary aim was to determine the effect of early azathioprine versus late azathioprine initiation in combination with EEN induction therapy on sustained steroid-free clinical remission at 6 and 12 months.

## Methods

### Patients

This was a retrospective study conducted at a tertiary paediatric hospital in Singapore. A prospectively maintained hospital database of children with inflammatory bowel disease was used to identify newly diagnosed children with CD from 2011 to 2014. The case records were reviewed and relevant data including demographics, details of disease location, serial weight and height measurements, and details of standard inflammatory markers such as erythrocyte sedimentation rate (ESR), C-reactive protein (CRP), haemoglobin, platelets, and albumin were extracted. The diagnosis of CD was based on standard criteria including clinical, endoscopic, histological and radiological findings [19]. Each patient had previously undergone oesophagogastroduodenoscopy, ileocolonoscopy, and a histological analysis of mucosal biopsies from multiple sites. Small bowel imaging with magnetic resonance imaging enterography is routinely performed for all newly diagnosed cases of CD as part of the standard protocol at our centre. The disease phenotype was classified according to the Paris classification for paediatric inflammatory bowel disease [20].

Disease activity was ascertained using the Paediatric Crohn’s Disease Activity Index (PCDAI) score [21]. PCDAI scores were calculated at diagnosis, at the end of the EEN therapy, and during routine visits at the specialized inflammatory bowel disease (IBD) clinics at 6 months and 12 months. Remission was defined as a PCDAI score of ≤10, while relapse was defined as a PCDAI score of > 10. All patients diagnosed with CD were routinely started on 5-aminosalicylic acid at our centre.

The inclusion criteria for the study were: (i) newly diagnosed children aged less than 18 years with active CD, (ii) completion of 8 weeks course of EEN as induction therapy, and (iii) a minimum follow-up period of one year following completion of the EEN course. Patients who had been previously treated with corticosteroids, immunosuppressive medications or biologics were excluded. This study was approved by the hospital institutional review board.

### Data collection

Data were collected from the patients’ medical, dietetic and laboratory records. Anthropometric records (height and weight) and PCDAI scores were collected at diagnosis, at approximately 8 weeks (post-EEN) and at 6 and 12 months post-EEN therapy. Height, weight and body mass index (BMI) measurements were converted into standard deviation z-scores using an age and sex-matched population as a reference standard [22]. Systemic markers of disease activity, namely, erythrocyte sedimentation rate (ESR), C-reactive protein (CRP), haemoglobin, platelets, and albumin were collected at diagnosis and post-EEN. Medication history including thiopurines, aminosalicylic acid, corticosteroid and other immunosuppressant use were also recorded.

### Exclusive enteral nutrition protocol

All patients recruited were started on EEN using whole casein polymeric milk formula (Modulen IBD, Nestle, Nunspeet, the Netherlands) as an induction therapy to complete a course of 8 weeks. They were referred to a dietician prior to initiation of EEN. Daily volumes of formula were prescribed based on the child’s estimated energy requirement (EER). Each child’s EER was calculated using the Schofield equation, which estimates basal metabolic rate based on body weight [23]. EEN was initiated with an incremental increase in milk volume over 2 to 3 days to avoid intolerance of the formula and refeeding syndrome. Patients were reviewed regularly and, when required, energy intake was modified by adjusting formula volumes based on the adequacy of weight gain. During the period of EEN, sugar-free chewing gum, boiled sweets, and water were allowed. A normal diet was reintroduced gradually after completion of the therapy, starting with low allergen food and gradually increasing over 2 weeks, while incrementally decreasing the amount of enteral formula.

### Maintenance therapy with Thiopurine use

There were two groups of patients in this cohort: those who received azathioprine early (within one month of diagnosis) were classified as the Early Azathioprine (EAZ) group while those who were started on Azathioprine only following a relapse were classified as the Late Azathioprine (LAZ) group. As our centre did not have a standardized protocol regarding thiopurine therapy, initiation of azathioprine was solely determined by each clinician’s treatment decision. Azathioprine was normally prescribed at a dose of 2–2.5 mg/kg per day. The clinical characteristics, PCDAI score and laboratory parameters were further compared between the two groups of patients.

All patients were monitored over a 12-month period following the EEN therapy to determine the duration of remission. Remission on azathioprine was defined as a PCDAI score of ≤10 without requiring any steroid therapy, further courses of EEN therapy, other immunosuppressant or biologic therapy.

### Statistical analyses

Statistical analysis was performed using IBM SPSS Statistics Version 22. Continuous variables are presented as the means (±SD). Non-continuous data are expressed as percentages. Comparisons of quantitative paired data and unpaired data were performed using a paired sample t-test and the Mann-Whitney U Test, respectively. Durations of remission were compared using Kaplan-Meier survival analysis, and a Cox regression model was used to adjust for potential confounders for comparisons between the EAZ and LAZ groups. A *p-*value of < 0.05 was regarded as statistically significant.

## Results

### Baseline characteristics of the study patients (Table 1 and Fig. 1)

Forty newly diagnosed children with CD from 2011 to 2014 were started on an EEN induction therapy of which 33 patients completed the treatment course. All patients were of SEA origin. Most of the patients (70%) had ileocolonic disease (L3). Almost a quarter of the patients had mainly colonic involvement (L2). Approximately 40% of the cohort had upper gastrointestinal tract involvement. The majority of patients (85%) had non-stricturing disease (B1) while three patients had stricturing disease (B2) and two had stricturing and penetrating (B2B3) disease. The mean follow-up period was 19.4 ± 6.8 months. Seven patients were unable to tolerate the EEN course and were switched to corticosteroids for induction therapy. All children received EEN orally, and none required nutrition via a nasogastric route.

**Table 1:** Demographics and Baseline Disease characteristics of children with Crohn’s Disease, *n* = 33

Demographics	n (%) or Mean ± SD or Median (range)		
Gender			
Males	22 (67%)		
Females	11 (33%)		
Race			
Chinese	14 (43%)		
Malay	6 (18%)		
Indian	10 (30%)		
Others	3 (9%)		
Median Age at presentation, years	13y (range 3–17)		
BMI z-score	−1.38 ± 1.57		
Disease location			
L1 terminal ileum	2 (6%)		
L2 colon	8 (24%)		
L3 ileo-colonic	23 (70%)		
L4, upper GI	13 (39%)		
Disease behavior			
B1 Non-stricturing	28 (85%)		
B2 Stricturing	3 (9%)		
B3 Penetrating	0 (0%)		
B2B3 Stricturing and Penetrating	2 (6%)		
Perianal involvement	4 (12%)		
Clinical parameters	Pre-EEN	Post-EEN	*p*-value
PCDAI baseline	32.7 ± 9.2	4.2 ± 5.1	< 0.0001
BMI z-score	−1.38 ± 1.57	− 0.82 ± 1.27	0.004
Weight (kg)	36.9 ± 14.36 kg	38.18 kg ± 12.62	< 0.001
Weight z-sore	−1.31 ± 1.75	− 0.84 ± 1.32	0.0001
Height (cm)	147.1 ± 18.8 cm	149.2 cm ± 18.72	0.009
Height z-score	− 0.58 ± 1.45	0.39 ± 1.49	0.009
Hemoglobin g/dL	11.4 ± 1.5	12.2 ± 1.5	0.008
Albumin g/L	30.7 ± 7.5	38.7 ± 3.9	< 0.0001
Platelets ×10^9^	464 ± 161	370 ± 111	< 0.0001
ESR mm/h	51.6 ± 30.1	13.3 ± 7.1	< 0.0001
CRP mg/L	44.6 ± 51.0	5.2 ± 7.6	0.001

*PCDAI* Paediatric Crohn’s Disease Activity Index, *BMI* Body Mass Index, *ESR* Erthrocyte Sedimentation Rate, *CRP* C-Reactive Protein

**Fig. 1:**
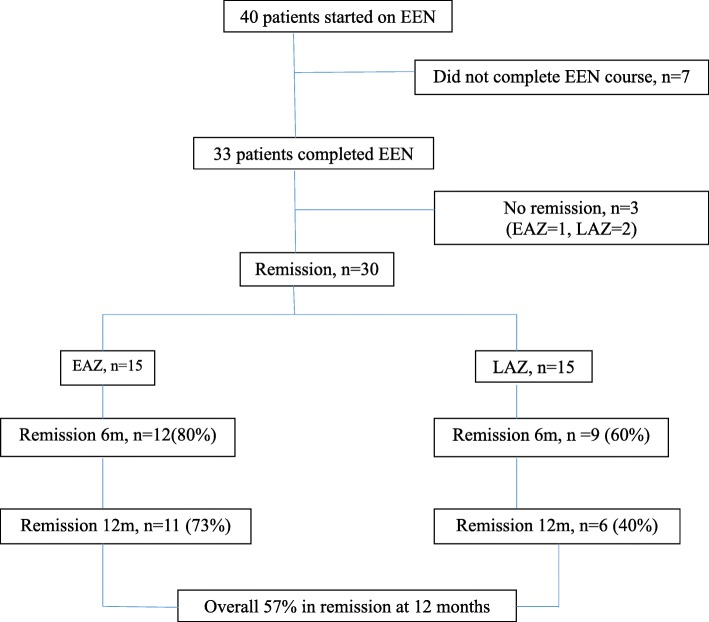
Remission Rates of Early AZA versus Late AZA group at 6 and 12 months

### Response to EEN induction

Of the 33 children (82.5%) who completed the EEN induction, 91% (30/33) achieved remission (PCDAI ≤10). There were significant improvements in growth, PCDAI and biochemical activity disease markers (CRP, ESR, albumin, platelet count and haemoglobin) post-induction with EEN (Tables 1 and 2).

**Table 2:** Comparison of height z-score, weight z-score and BMI z-scores: pre and post EEN, pre and 6 months post EEN*, pre and 12 months post EEN^#^

	Pre-EEN	Post-EEN	*p*-value	6 m post EEN	*p*-value^*^	12 m post EEN	*p*-value^*#*^
Weight Z-score	−1.31 ± 1.75	−0.84 ± 1.32	0.0001	0.043 ± 2.02	0.002	−0.27 ± 1.18	< 0.0001
Height Z-score	−0.58 ± 1.45	0.39 ± 1.49	0.009	−0.08 ± 1.22	0.004	−0.25 ± 1.17	0.58
BMI Z-score	−1.38 ± 1.57	−0.82 ± 1.27	0.004	−0.41 ± 1.25	0.001	−0.16 ± 1.3	0.001

*EEN* exclusive enteral nutrition, *m* months

*pre EEN and 6 months post EEN; ^#^pre EEN and 12 months post EEN

For the remaining 3 patients who did not achieve remission, all had B2 disease. There were some improvements in inflammatory markers: CRP reduced from a mean of 16 ± 15.5 mg/L to 10 ± 12.1 mg/L; albumin increased from 21 ± 16.3 g/L to 34 ± 2.6 g/L; and haemoglobin increased from 10.3 ± 2.5 g/dL to 12.7 ± 1.6 g/dL. However, ESR increased from 17 ± 14.2 mm/h to 22 ± 12.6 mm/h. Two patients with B2B3 disease achieved an initial remission with EEN therapy but relapsed after 2.5 and 8 months respectively. Four children had perianal disease; three of them achieved remission with EEN. The last patient, who failed to achieve remission, had both B2 and perianal disease.

At diagnosis, 23% of the patients were thin (BMI z-score ≤ − 2 SD) with a mean body mass index (BMI) z-score of − 1.4 ± 1.5 (Table 2). Post EEN, the mean BMI z-score improved to − 0.82 ± 1.27 (*p* < 0.001). Similarly both the weight and height z-scores also showed significant increase post EEN. The effects on growth appeared to be sustained over 6 months post-EEN with all the three parameters showing significant improvements. Similarly at 12 months post-EEN, both weight and BMI z-scores continued to show significant improvements but not height z-score.

### Azathioprine therapy

Oral azathioprine was started within a month of diagnosis in 16 out of 33 (49%) patients, composing the EAZ group (Fig. 1). In the LAZ group, which consisted of 17 (51%) patients, azathioprine was started only following a relapse at a mean of 5.7 (range 3–9) months post EEN. The demographic and clinical characteristics, including the PCDAI score, of EAZ and LAZ were comparable at baseline between the 2 groups (Table 3). Haemoglobin, albumin, platelet count and CRP did not show any significant difference. However, ESR was significantly higher in the EAZ group compared to the LAZ group (63.2 ± 37.5 mm/h vs 41.3 ± 16.9 mm/h, *p* = 0.03). Overall, none of the patients who received azathioprine experienced hepatotoxicity, marrow suppression or malignancy during the study period.

**Table 3:** Comparison of baseline demographics and clinical parameters of patients in Early Azathioprine (EAZ) versus Late Azathioprine (LAZ) groups

Demographics	EAZ (*n* = 16)	LAZ (*n* = 17)	*p* value
	N (%) or mean ± SD or median (range)		
Gender			
Males	12 (54.5%)	10 (45.5%)	0.32
Females	4 (36.4%)	7 (63.6%)	0.38
Race			
Chinese	7 (50%)	7 (50%)	
Malay	1 (16.7%)	5 (83.3%)	
Indian	6 (60%)	4 (40%)	
Others	2 (66.7%)	1 (33.3%)	
Median Age at presentation in years and (range)	11.5 (3–16)	14 (4–17)	0.18
Clinical parameters			
BMI z-score	−1.12 ± 0.9	−1.5 ± 1.9	0.43
PCDAI baseline	32.1 ± 8.3	33.1 ± 10.3	0.77
Hemoglobin g/dL	11.3 ± 1.2	11.4 ± 1.7	0.80
Albumin g/L	30.6 ± 6.1	30.7 ± 8.6	0.97
Platelets × 10^9^	471 ± 175	456 ± 150	0.79
ESR mm/h	63.2 ± 37.5	41.3 ± 16.9	0.03
CRP mg/L	42.1 ± 40.3	47.1 ± 61.3	0.79

*PCDAI* Paediatric Crohn’s Disease Activity Index, *BMI* Body Mass Index, *ESR* Erthrocyte Sedimentation Rate, *CRP* C-Reactive Protein

### Maintenance of remission

Thirty patients achieved remission; half (*n* = 15) were from the EAZ group. At 6 months post-EEN, 80% (*n* = 12) of the patients from the EAZ group were in remission versus 60% (*n* = 9) from the LAZ group (*p* = 0.018). By 12 months, 73% (*n* = 11) of the EAZ patients were still in continuous remission with no relapse in the intervening period compared to 40% (*n* = 6) from the LAZ group (*p* = 0.57). The overall remission rates at 6 and 12 months were 70 and 57%, respectively (Fig. 1).

**Fig. 2:**
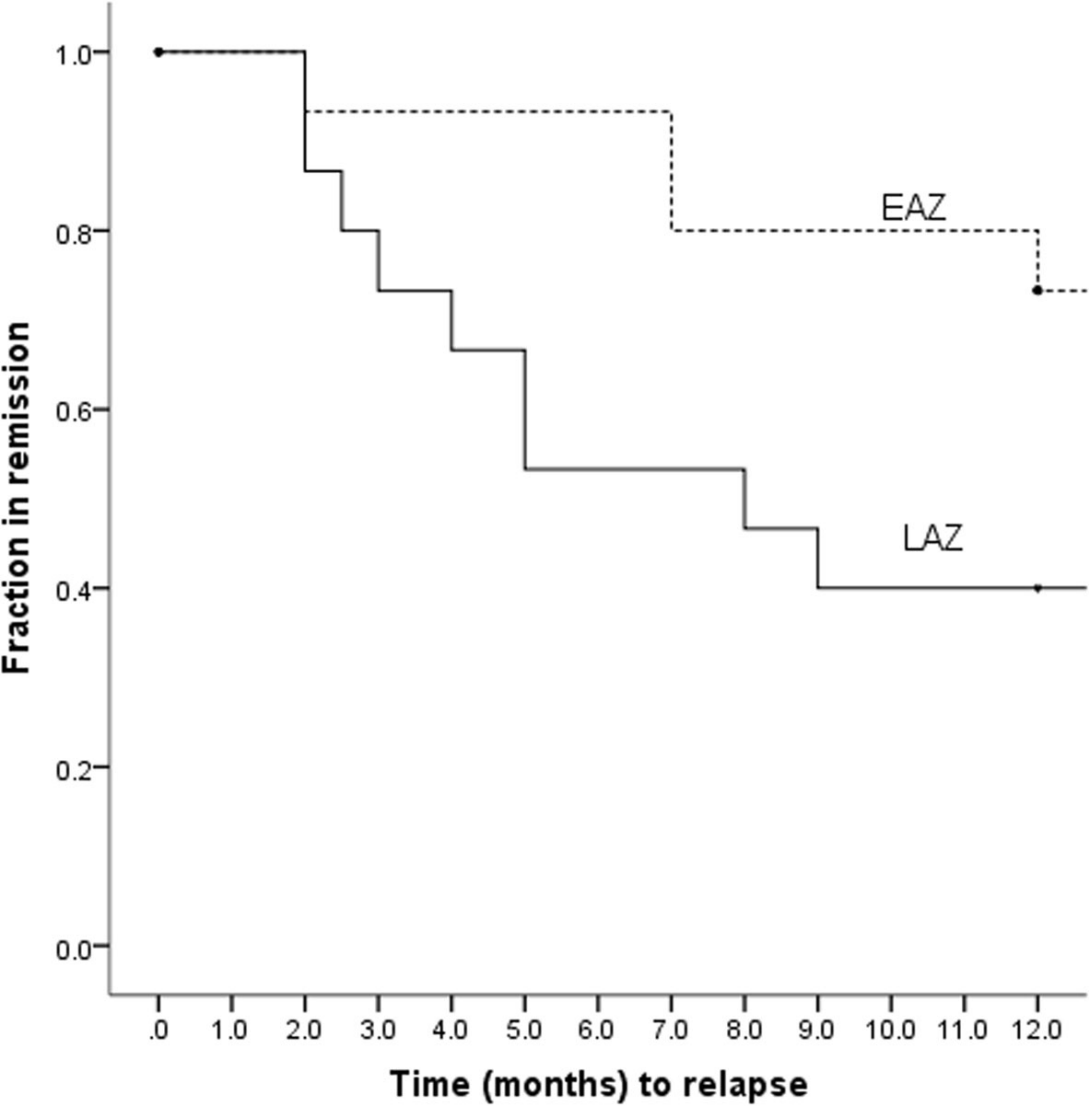
Kaplan-Meier survival analysis for relapse-free duration of remission of Early Azathioprine (EAZ) versus Late Azathioprine (LAZ) groups. *EEN* Exclusive Enteral Nutrition. *EAZ* Early Azathioprine. *LAZ* Late Azathioprine. *m* months

We further addressed the remission rate in a Kaplan-Meier survival model (Fig. 2). Remission was maintained longer in the EAZ group compared to LAZ with a mean time to relapse of 10.1 (3.5) months vs 7.9 (4.1) months, respectively (*p* = 0.048). The multivariate hazard ratio determined by the Cox regression analysis was 1.9 (95% CI, 0.4–7.5, *p* = 0.34) after adjusting for age, sex, disease site, and baseline PCDAI score (Table 4).

**Table 4:** Hazard ratio and their 95% Confidence Interval of occurrence of relapse for Early Azathioprine (EAZ) versus Late Azathioprine (LAZ) groups

	EAZ	LAZ	*p*-value
Number of cases	15	15	
Unadjusted HR (95% CI)	1 (reference)	3.1 (1.0–10)	0.06
Adjusted for age and gender HR	1 (reference)	2.2 (0.6–8.1)	0.23
Multivariate HR (95% CI)^a^	1 (reference)	1.9 (0.4–7.5)	0.34

^a^Adjusted for age, gender, disease site, and PCDAI at baseline

*HR* Hazard Ratio

## Discussion

Phenotypes of IBD in South Asian children have been reported to be more complicated than in Caucasians, with higher rates of poor weight gain, fistula formation, and requirements for immunosuppressive therapy [24]. Korean children with CD have more prevalent small bowel disease and perianal fistulas compared with European populations [25]. Environmental and genetic factors most likely account for the observed phenotypical differences [26]. Asian patients with IBD have different susceptibility genes compared to their Caucasian counterparts. [27]. They also have genetic mutations that vary from those previously associated with IBD in Caucasians. JW1 mutation, a novel *NOD2* mutation have been reported in Malaysians with CD and P268S mutations in Han Chinese and Indians [28]. Environmental factors, notably diet and antibiotics usage, also play an integral role in the pathogenesis of IBD, but these influences appear to vary in different regions [26].

EEN is a well-established and effective induction therapy for children with CD with remission rates comparable to corticosteroids [29]; however, it has not been universally adopted in paediatric centres including those in Asian/SEA countries. Poor patient compliance, cultural factors, costs of the formulas and a lack of physicians’ experience are possible contributory factors for the low implementation of EEN therapy.

To our knowledge, this study is the first to report the efficacy of EEN in inducing remission in an SEA cohort of children with newly diagnosed CD. Our results indicated a high response rate of 91%, comparable to published western reports [8, 29, 30]. In concordance with other studies [6, 29, 31], there were significant improvements in PCDAI, inflammatory markers (albumin, platelets, ESR, and CRP), and growth parameters after 8 weeks of EEN. Interestingly, the patients who did not achieve a remission were those with stricturing disease (B2) at presentation, suggesting the limited efficacy of EEN in this group.

Contrary to the reports of an aggressive nature of CD in Asian phenotypes compared with Caucasians, our data showed a comparable response rate with EEN therapy in our cohort. Despite genetic variations among different ethnicities, these differences were unlikely to influence the response to EEN since the therapeutic mechanisms postulated are a modulation of the intestinal microbiome, bowel rest through the reduction in antigenic load and direct anti-inflammatory effects [11, 12].

Although the majority of patients achieve a clinical and biochemical remission following EEN induction, the reported relapse rate within 12 months is high, ranging from 50 to 80% [32]. Our cohort showed overall remission rates of 70 and 57% at 6 and 12 months, respectively. Early azathioprine initiation was the only identifiable factor that influenced the duration of remission, with a remission rate of 73% at 12 months compared to 40% in the LAZ group. We did not find any significant association with age of onset, disease site, sex, or PCDAI score at diagnosis.

There were also significant improvements in growth parameters at 6 and 12 months post EEN therapy. These findings suggest that the growth benefits (particularly weight and BMI z-scores) of EEN induction therapy may extend for at least 12 months post-EEN.

Population studies on the effectiveness of thiopurines in maintaining steroid-free remission have not consistently shown benefit: some reported excellent efficacy while others recorded nil to modest results. In a randomized control trial evaluating children with moderate to severe Crohn’s disease, Markowitz et al. reported that more than 90% of the patients treated with steroids and 6-mercaptopurine (6MP) at diagnosis were able to maintain remission to at least 18 months [16]. Our study demonstrated a similar high remission rate of 73% at 12 months for the EAZ group.

Another retrospective study reported much lower remission rates, of approximately 40% at 1 year [33]. The method of induction therapy for their group of patients was heterogeneous, including enteral nutrition, steroids, and 5-aminosalicylic acid derivatives*.* Boyle et al. reported remission rates of 65 and 42% at 6 and 12 months, respectively, in a ‘real-world’ clinical setting [34]. However, the study did not mention induction methods or the timing of thiopurine initiation. Frivolt et al. observed a high relapse rate of 67% at 12 months despite early azathioprine initiation within one month following EEN induction therapy [15]. Their cohort included both newly diagnosed and relapsed cases, which may have adversely affected the results. Both Markowitz [16] and our study included only treatment naïve patients with newly diagnosed CD.

Mucosal healing is the ultimate therapeutic goal because it predicts a sustained clinical remission and resection-free survival in patients with CD [35–37]. The use of biologics favouring the top-down approach to management is increasingly adopted by gastroenterologists [38]. However, the cost of biologics remains prohibitive for most patients, particularly in countries where there is a lack of central funding. In our experience, many patients continue to have reservations regarding starting biologics due to costs and perceived side effects, particularly those who are in clinical remission but have not achieved mucosal healing. Thiopurines continue to be the mainstay of maintenance therapy for children with CD in the ‘real-world’ clinical setting.

## Conclusion

In conclusion, this is the first report to demonstrate that EEN is a highly effective therapy for newly diagnosed CD in SEA children. Concomitant early thiopurine, particularly when initiated within a month of diagnosis, is effective in maintaining steroid-free clinical remission in the majority of patients for at least one year. We recognize that this study has inherent limitations due to its retrospective nature and limited sample size. Future research should focus on obtaining a larger sample size, determination of the degree of mucosal healing, and collecting data over a longer period performed in a prospective manner.

## Abbreviations

CD: Crohn’s Disease
CRP: C-Reactive Protein
EAZ: Early Azathioprine
EEN: Exclusive Enteral Nutrition
ESR: Erythrocyte Sedimentation Rate
IBD: Inflammatory Bowel Disease
LAZ: Late Azathioprine
PCDAI: Paediatric Crohn’s Disease Activity Score
SEA: South East Asia
